# Pharmaceutical Assessment of Watermelon Rind Pectin as a Suspending Agent in Oral Liquid Dosage Forms

**DOI:** 10.1155/2022/9526404

**Published:** 2022-11-07

**Authors:** Frederick William Akuffo Owusu, Mariam El Boakye-Gyasi, Marcel Tunkumgnen Bayor, Christina Osei-Asare, Raphael Johnson, Yaa Asantewaa Osei, Victoria Agyeibea Asare, Kezia Akoley Mensah, Prince George Acquah, Desmond Asamoah Bruce Otu, Rocklyn Asante

**Affiliations:** ^1^Department of Pharmaceutics, Faculty of Pharmacy and Pharmaceutical Sciences, Kwame Nkrumah University of Science and Technology, Kumasi, Ghana; ^2^Department of Pharmaceutics, School of Pharmacy, Central University, Miotso, Ghana

## Abstract

Pectin is a high molecular weight polymer which is present in virtually all plants where it contributes to the cell structure. Pectin is a high valuable food ingredient widely used as a gelling agent and thickening agent with limited use in the pharmaceutical industry. The objective of this study is to evaluate the suspending properties of pectin from watermelon rind. Tragacanth was used as a standard suspending agent to which the suspending properties of pectin from watermelon rinds were compared with. The extracted pectin was subjected to phytochemical and physiochemical characterization for its safety and suitability to use as a suspending agent. Paracetamol suspensions were formulated using tragacanth concentrations of 0.5% w/v, 1% w/v, and 2% w/v and compared with paracetamol suspensions containing the same concentrations of watermelon pectin. The suspensions were all tested for their pH, sedimentation rate, sedimentation volume, flow rate, and ease of redispersibility over a period of 4 weeks. At the end of the 4-week period, all formulated suspensions had no changes in their pH values. Suspensions containing the extracted pectin had a lower rate of sedimentation and ease of redispersibility compared to that of tragacanth. In addition, their sedimentation volumes as well as flow rates were comparable to that of the tragacanth formulations. Ultimately, pectin from watermelon rind can serve as a suitable alternative to tragacanth in formulation of pharmaceutical suspensions.

## 1. Introduction

Watermelon (*Citrullus lanatus*) is a tropical fruit which belongs to the Cucurbitaceae family and is widely cultivated around the world. The outer part of the fruit which is known as the rind is greenish in colour and has been reported to contain complex polysaccharides such as pectin [1–3]. As watermelon rind represents about one third of the total fruit mass, researchers have been trying to discover new ways to utilize it, including harnessing pectin from it [2, 3]. Pectin is a polysaccharide that contains mainly galacturonic acid joined by *α*-(1, 4) linkages. In the food and pharmaceutical industries, pectin is widely used due to its hydrocolloid properties. It is widely used as an additive in the food industry [4]. It can function as a stabilizer in confectionery products such as yogurt drinks and milk; a thickener in bread, frozen dough, and yogurt; an emulsifier for cream, milk, and ice cream; and a gelling agent for jam and jellies [5].

A significant percentage of the total watermelon crop grown in Ghana (20-40%) is wasted each year due to the inability to sell the total yields within the peak growing season, especially when food quantities are reached [6, 7]. This waste is as a result of the inadequate knowledge on storage since many producers harvest their melon crop at most twice in a growing season. Also, a large number of farmers have lots of difficulty in selling, processing, and transporting these watermelons. All of these factors contribute to the nearly 30% of the watermelon crop that goes unharvested each year [6–8]. This wasted crop represents a significant potential for the development of value-added products such as pectin from the watermelon rind which would have hitherto gone to waste and can lead to further diversification of the watermelon rind for economic value.

The currently used suspending agents from synthetic and inorganic sources are fronted with challenges such as cost, availability, sustainability, and compatibility [9, 10]. There is, therefore, the need to find other alternative suspending agents that are from natural sources and are readily available. These alternative suspending agents should also be inexpensive which will in effect lower the total cost incurred in the production of the suspension. They should also be nontoxic and moreover not pose any irritations [9–12]. This study therefore seeks to evaluate the suspending properties of pectin from watermelon rind. Its suspending properties if found comparable to standard suspending agents can serve as a good option in pharmaceutical preparation of suspensions and ultimately reduce the cost of producing liquid pharmaceutical dosage forms for local manufacturers in Ghana.

## 2. Materials and Methods

### 2.1. Materials

Watermelon rind, benzoic acid (BDH England), tragacanth (Sigma Aldrich, Darmstadt Germany), paracetamol powder (purity: 99.5%), chloroform (BDH England), distilled water, and ethanol (96%). All other reagents used were of analytical grade.

### 2.2. Method

#### 2.2.1. Sample Collection and Drying

The watermelon fruit was purchased from retailers at a local market. The fresh watermelon was washed and with the aid of a knife, the rind was removed from the flesh. The peel was then leached with tap water to remove soluble solid. The watermelon rind was cut into smaller pieces and dried under the sun for 2 weeks. The dried watermelon rind was blended using a domestic lab blender into very fine powder.

#### 2.2.2. Extraction of Pectin from Dried Watermelon Peels Using the Water-Based Extraction Method

A mass (200 g) of the powder was weighed into a beaker. It was then treated with 12 L of acidified water of pH 2 and placed in a water bath set at temperature of 70°C. The assembly was run for a 3-hour duration. The sample was cooled and filtered with a muslin cloth. 10 L of 96% ethanol was added to the filtrate to facilitate precipitation of pectin. The solution was centrifuged at 400 rmp for 15 minutes to separate the pectin and was dried under vacuum at 50°C. Pectic substances were ground using mortar and pestle and weighed [11].

### 2.3. Characterization of Pectin

#### 2.3.1. Pectin Yield

The pectin yield was determined as described by Kamm B and Kamm M [13]. An analytical balance was used to determine the weight of extracted watermelon rind pectin (W1) and powdered rind (W2). The percentage yield was then calculated using the formula:

(1) $$\begin{array}{c} \%\text{Yield}=\frac{\text{weight of pectin extracted }\left(W1\right)}{\text{weight of powdered rind }\left(W2\right)}\times 100. \end{array}$$

#### 2.3.2. Proximate Composition Analysis

The watermelon pectin was analysed for moisture content, ash content, crude protein, crude fat, crude fibre, and total carbohydrate following standard techniques [14, 15].

### 2.4. Elemental Content

The elemental contents were determined in the watermelon pectin with an atomic absorption spectrophotometer (Buck Scientific Model 210 V GP) as previously reported in [16, 17].

#### 2.4.1. Fourier Transformed Infrared (FTIR) Spectroscopy

FTIR analysis of watermelon pectin was performed with an FTIR spectrometer (PerkinElmer, UATR Spectrum 2, 941333, UK). The esterification degree (DE) of the sample was calculated by determining the peak area values of the free carboxyl groups (1709.55 cm^−1^) and the esterified groups (1585.84 cm^−1^) by the following equations [18].

(2) $$\begin{array}{c} \text{DE}=124.7\times R+2.2013,\\ R=\frac{A_{1709.55}}{A_{1709.55}+A_{1585.84}}\times 100, \end{array}$$

where DE is the degree of esterification and *A*_1709.55_ and *A*_1585.84_ are the absorbance densities at 1709.55 cm^−1^ and 1585.84 cm^−1^, respectively [19].

### 2.5. Phytochemical Tests

The extracted pectin was tested for the presence of some phytochemicals such as saponins, flavonoids, tannins, and glycosides [20].

#### 2.5.1. Test for Saponins

On a water bath, the powdered pectin was boiled with distilled water. It was filtered while it was still hot; then, the filtrate was diluted with distilled water and rapidly agitated for 2 minutes. Saponins were detected by the formation of a persistent foam [20].

#### 2.5.2. Test for Flavonoids

The aqueous pectin extract was placed into a test tube along with a piece of filter paper, which was then allowed to dry. The paper was then immersed into concentrated ammonia. The presence of flavonoids is confirmed by the appearance of a bright yellow colour [20].

#### 2.5.3. Test for Tannins

In a test tube, 1% lead acetate drops were added to an aqueous extract of watermelon pectin, and the development of a precipitate was observed. The presence of tannins was revealed by the development of precipitate [20].

#### 2.5.4. Test for Glycosides

The pectin sample's filtered water extract was heated with weak sulphuric acid (10%) before being filtered a second time. To make the filtrate alkaline, two drops of sodium hydroxide were added. The Fehling's solutions A and B were then combined and heated in a water bath. The resulting mixture was examined for a red precipitate [20].

### 2.6. Identification Tests

Identification tests were performed on the extracted pectin as previously described [21, 22].

### 2.7. Preparation of Paracetamol Suspension

The paracetamol suspensions (2.4 0% w/v) were prepared using pectin concentrations of 0.5% w/v, 1.0% w/v, and 2.0% w/v each using chloroform water (D/S) and benzoic acid (0.10%) as preservatives. The formula for formulating the suspensions is given in Table 1. Only the tragacanth and pectin (suspending agent) concentrations were varied in the formula [23]. The suspensions were prepared using the method of doubling the bulk for powders in a mortar. A slurry was subsequently formed with the aid of water, and the preservatives were added. The required volume of water was then added, and the resultant mix was homogenised at 20000 rpm with an electronic homogeniser (Drawell D-160) for 5 minutes to obtain a homogenous mixture. The formulated suspension was then transferred into an amber bottle. This procedure was used in preparing all suspensions.

### 2.8. Quality Control Tests on Formulated Suspensions

#### 2.8.1. pH

The pH of each of the formulated suspensions was measured when freshly prepared and at weekly intervals. The determinations were done in triplicates, and their means and standard deviations were recorded [23–25].

#### 2.8.2. Sedimentation Rate and Volume

The sedimentation rates were determined by measuring the level of sediment in the measuring cylinder at intervals of ten (10) minutes for sixty (60) minutes. The sedimentation volume of the suspension was determined by measuring the volume of the sediments in 50 mL of the formulated suspension, on weekly basis for 4 weeks. The sedimentation volume (*F*) was calculated using the formula *F* = Vu/Vo (1) where Vu is the ultimate volume of sediment and Vo is the original volume of sediment before settling occurred. Triplicate determinations were done, and the mean was calculated [23–25].

#### 2.8.3. Viscosity and Flow Rate

The viscosity of all suspensions was determined using a Drawell digital viscometer (HBDV-I) fitted with an RV spindle No. 2. Using a stopwatch, the time required for the formulated suspensions to flow through a 20 mL pipette was determined. The process was repeated three times, and their means and the standard deviations were recorded [23–25].

#### 2.8.4. Redispersibility

The 50 mL formulated suspensions with different concentrations of both tragacanth and pectin were evaluated for redispersibility at weekly intervals for 4 weeks, by turning it through a 180° cycle. Redispersibility was recorded as the number of inversions (strokes) required to completely resuspend the formulations [23–25].

## 3. Results and Discussions

### 3.1. Characterization of Extracted Pectin

The amount of pectin obtained (Table 2) from the watermelon rind was similar to the yields reported by centrifugation (14.3%) and higher than that reported from cheesecloth methods (10.6%) indicating that an efficient extractive procedure was used [26]. The proximate composition obtained (Table 2), which are essential factors in the utilization and storage of pectin, showed low values when compared to standards [27, 28]. The low moisture content indicates that the extracted pectin can be safely stored for a longer duration with lower susceptibility to microbial growth while the low ash content indicates lower levels of inorganic impurities, an indication that the watermelon pectin was of satisfactory quality [28, 29]. The high total carbohydrates compared to the crude protein and fat indicate that carbohydrates are the highest calorie contributor [30].

The elemental analysis (Table 3) shows the presence of essential minerals needed for biochemical processes (P, K, Ca, Mg, Na, Fe, and Cu). The toxic metals (Pb, Cd, and Hg) were present in very low amounts suggesting the possible nontoxicity of the watermelon pectin and thus could be employed as a pharmaceutical excipient [31, 32].

**Figure 1:**
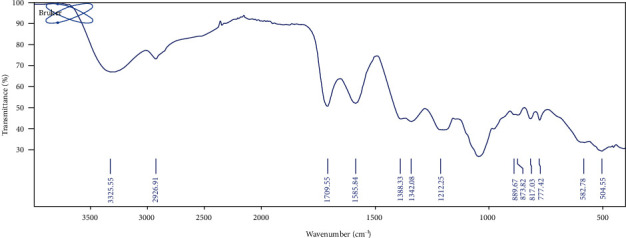
FTIR spectra of watermelon pectin.

The analysis of the FTIR spectra of the extracted watermelon pectin depicted a high degree of similarity when peak positions were compared with commercial rapid-set pectin and reported analogous works on pectin from watermelon rind [28, 33–35] (Figure 1). The presence of intra- and intermolecular hydrogen bonding, C-H and *C* = *O* stretching vibrations of esterified carboxyl groups, and free carboxyl groups was observed at spectra frequencies 1600-1300, 2926.91 cm^−1^, and 1709.55 cm^−1^, respectively [28, 33, 34]. Moreover, the weak peaks (889.67-504.55 cm^−1^) are linked possibly to the *α*- and *β*- configurations of pyranose cycles. These functional groups revealed that the extract obtained was pectin [28, 34, 35]. The identity of the extract was further corroborated as pectin due to their conformance to standard identification tests for pectin (Table 4) [21]. The watermelon pectin had a high degree of esterification (DE > 50) which was found comparable with reported values [19]. Phytochemical characterization of watermelon rind pectin powder revealed the presence of tannins and saponins and the absence of glycosides and flavonoids (Table 5).

### 3.2. Evaluation of Formulated Suspensions

#### 3.2.1. pH and Redispersibility

**Figure 2:**
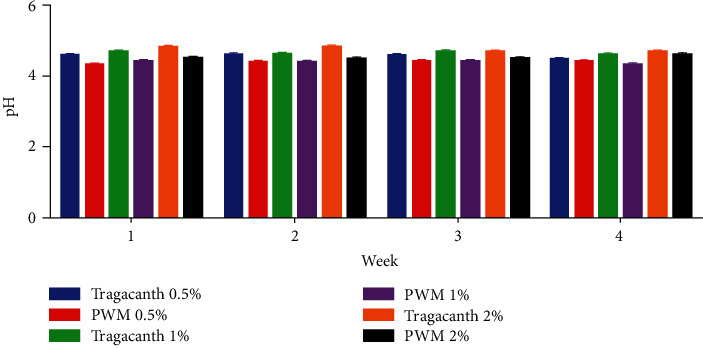
pH of tragacanth and pectin suspensions. PWM-pectin from watermelon rind.

**Figure 3:**
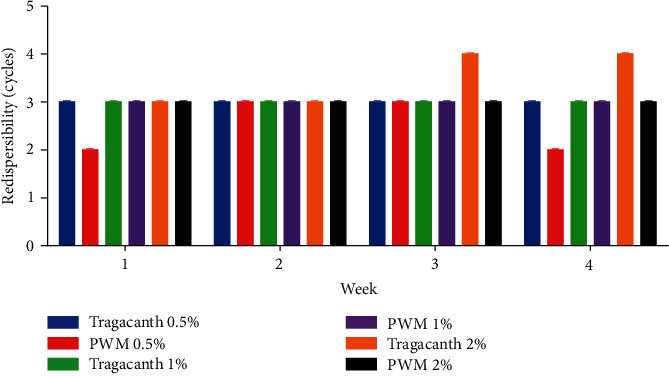
Redispersibility of tragacanth and pectin suspensions. PWM-pectin from watermelon rind.

pH of the suspension is one of the most important factors to be considered in the stability of products. From the study, the pH of the various suspensions was all weakly acidic (Figure 2). Since over the 4-week period, the pH of all formulations remained fairly constant and within specifications [36, 37]. It can be inferred that upon storage, there would not be pH instabilities which in effect can prevent degradation of the suspension and enhance stability of the product. With respect to their ease of redispersibility, the concentration of tragacanth suspension between 0.5% w/v and 1% w/v was easily redispersed when rotated at an angle of 180°, but the higher concentration (2% w/v) was redispersed with little difficulty as it had 4 cycles for week 3 and 4. This can be attributed to tighter packing of the particles; therefore, addition of deflocculating agents such as potassium chloride or sodium chloride and aluminium sulfate may reduce the tight packing nature of the tragacanth suspension [38, 39]. With the pectin suspensions, all concentrations were easily redispersed without difficulty at 180° such that all concentrations had 3 cycles except for 0.5% w/v in the first and fourth weeks (2 cycles, respectively). Thus, the suspensions produced from pectin redisperse easily in comparison with suspensions produced from tragacanth (Figure 3).

#### 3.2.2. Rheology of Suspensions

**Figure 4:**
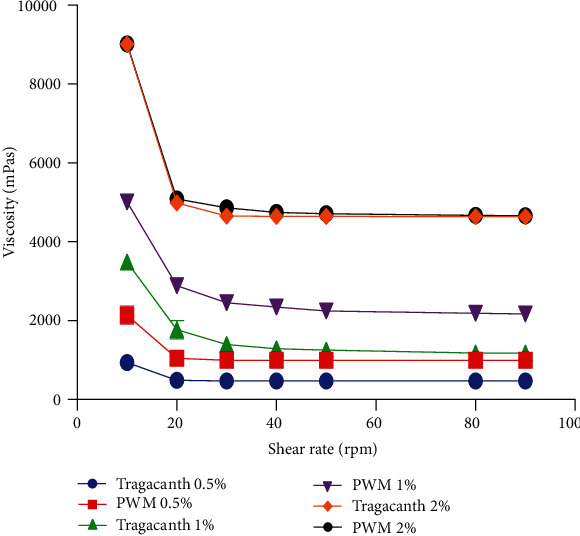
Viscosity of formulated suspensions. PWM-pectin from watermelon rind.

**Figure 5:**
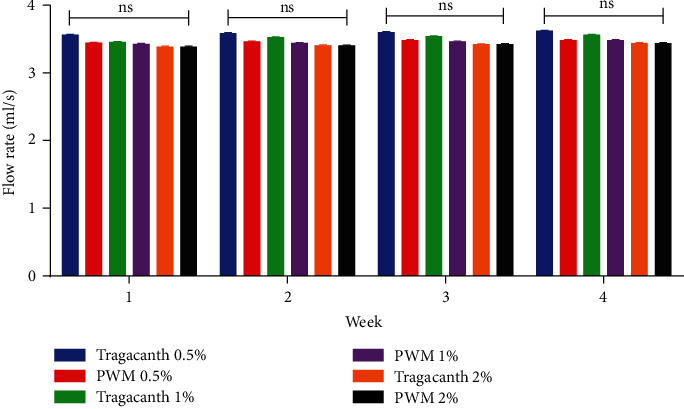
Statistical analysis on the flow rate of tragacanth and watermelon rind pectin suspensions using two-way ANOVA. *P* ≥ 0.05 not significant (ns). PWM-pectin from watermelon rind.

All formulated suspensions exhibited pseudoplastic flow which is required of an ideal suspension (Figure 4) [38, 39]. However, the viscosity of the watermelon rind pectin suspension was higher than that of the tragacanth suspensions at all concentrations used. Suspensions with higher viscosities are known to be relatively stable in comparison with less viscous suspensions [38–41]. Therefore, the higher viscosity of pectin from watermelon rind indicates its potential suitability as a suspending agent. It has been reported by Bamigbola et al. that as the concentration of a suspending agent is increased in a suspension, there is a resultant decrease in flow rate. This property is ideal in maintaining the pseudoplastic behaviour of the suspension and also ensuring that the suspension is easily pourable [42, 43]. The flow rate of the paracetamol suspension made with both tragacanth and pectin was found to be inversely proportional to their concentration with no statistical variations between the flow rate of suspensions prepared from tragacanth and watermelon rind pectin (Figure 5). This further corroborates the fact that pectin from watermelon rind has potential as a suspending agent with properties similar to that of tragacanth.

#### 3.2.3. Sedimentation Rate and Sedimentation Volume

**Figure 6:**
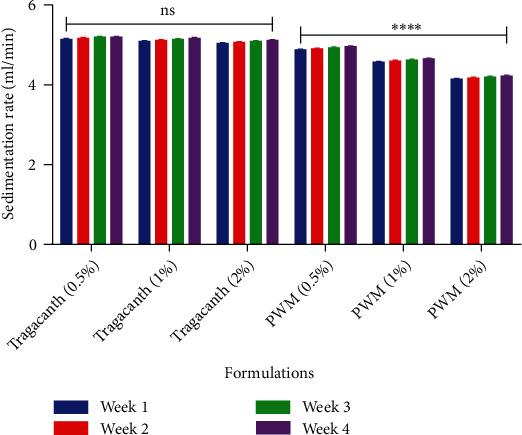
Statistical analysis on the sedimentation rate of tragacanth and watermelon rind pectin suspensions using two-way ANOVA multiple comparison test. ^∗∗∗∗^*P* ≤ 0.0001 and *P* ≥ 0.05 not significant (ns). PWM-pectin from watermelon rind.

**Figure 7:**
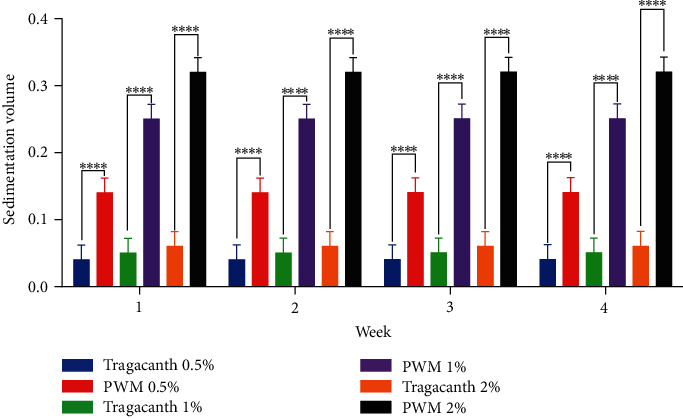
Statistical analysis on the sedimentation volume of suspensions containing tragacanth and watermelon rind pectin using student's two-tailed test. ^∗∗∗∗^*P* ≤ 0.0001.

After the formulation of pectin suspensions from watermelon rind, the rate of sedimentation decreased significantly (*P* < 0.05) with an increase in concentration as compared to that of the tragacanth suspension (*P* ≥ 0.05) (Figure 6). This goes to show that the suspending ability of pectin increases significantly (*P* ≥ 0.05) with an increase in concentration which produces a lower sedimentation rate. This lower sedimentation rate of suspensions formulated from watermelon rind pectin is a desirable attribute of an ideal suspension [38–41]. Sedimentation volume can have values ranging from less than 1 to 1. The larger the value, the better the suspendability property of the suspending agent [38, 40, 41]. The sedimentation volumes for suspensions prepared from watermelon rind pectin were significantly higher (*P* < 0.05) than that of tragacanth at all concentrations used (Figure 7). This clearly indicates the suitability of watermelon rind as a suspending agent.

## 4. Conclusion

Pectin obtained from watermelon rind was found to be comparable to tragacanth as a suspending agent and can therefore be utilized as such in pharmaceutical liquid dosage forms.

## Figures and Tables

**Table 1:** Formula for preparation of paracetamol suspension.

Ingredient	Quantities
Paracetamol powder	2.4 g
Benzoic acid (0.1% w/v)	0.1 g
Chloroform water (D/S)	50 mL
Suspending agent (1% w/v)	10 mL
Water to	100 mL

**Table 2:** Yield and proximate compositions of extracted pectin.

Parameter	Value
Pectin yield	14.1% w/w
Moisture content	11.5% w/w
Ash content	2.3% w/w
Crude protein	12.26% w/w
Crude fat	2.49% w/w
Crude fiber	0.10% w/w
Total carbohydrate	45.83% w/w

**Table 3:** Elemental content of extracted pectin.

Parameter	Value (mg/kg)
Mineral content	
P	5750.000
K	3630.000
Ca	1010.000
Mg	3790.000
Na	820.000
Heavy metals	
Fe	828.660
Cu	160.770
Pb	0.024
Cd	1.747
Hg	0.012

**Table 4:** Identification of watermelon rind pectin.

Test	Observation	Inference
Pectin solution + heating and cooling	A stiff gel was formed on cooling	Pectin present
Pectin solution + ethanol	A colourless gelatinous precipitate was formed	Pectin present
Pectin solution + NaOH (2 M)	A translucent gel was formed	Pectin present
Pectin gel + HCl (3 M) + boiling	White cotton-like precipitates is formed while boiling	Pectin present

**Table 5:** Phytochemical constituents in extracted pectin.

Test	Inference
Test for glycosides	Absent
Test for tannins	Present
Test for saponins	Present
Test for flavonoids	Absent

## Data Availability

The data used to support the findings of this study are included in the article and also available from the corresponding author upon request.

## References

[1] Lee K. Y., Choo W. S. (2020). Extraction optimization and physicochemical properties of pectin from watermelon (Citrullus lanatus) rind: comparison of hydrochloric and citric acid extraction. *Journal of Nutraceuticals and Food Science*.

[2] Jiang L. N., Shang J. J., He L. B., Dan J. M. (2012). Comparisons of microwave-assisted and conventional heating extraction of pectin from seed watermelon peel. *Advanced Materials Research*.

[3] Mendez D. A., Fabra M. J., Martínez-Abad A., Gorria M., López-Rubio A. (2021). Understanding the different emulsification mechanisms of pectin: comparison between watermelon rind and two commercial pectin sources. *Food Hydrocolloids*.

[4] Mohnen D. (2008). Pectin structure and biosynthesis. *Current Opinion in Plant Biology*.

[5] Vanitha T., Khan M. (2020). Role of pectin in food processing and food packaging. *Pectins-extraction, purification, characterization and applications*.

[6] Ministry of Food and Agriculture (MoFA), Ghana (2013). Food and agriculture sector; Vegetable production. http://mofa.gov.gh/site/?page_id=598.

[7] Ministry of Food and Agriculture, Ghana (2015). Agriculture in Ghana: Facts and Figures 2015. https://mofa.gov.gh/site/images/pdf/AGRICULTURE-IN-GHANA-Facts-and-Figures-2015.pdf.

[8] Danso-Abbeam G., Ehiakpor D. S., Aidoo R. (2018). Agricultural extension and its effects on farm productivity and income: insight from northern Ghana. *Agriculture & Food Security*.

[9] Mengesha Y., Tuha A., Seid Y., Adem A. A. (2021). Evaluation of Aloe weloensis (Aloeacea) mucilages as a pharmaceutical suspending agent. *Advances in Pharmacological and Pharmaceutical Sciences*.

[10] Deshmukh S. S., Katare Y. S., Shyale S. S. (2013). Isolation and evaluation of mucilage of Adansonia digitata Linn as a suspending agent. *Journal of Pharmaceutics*.

[11] Udonne J. D., Ajani O. O., Akinyemi M. L. (2016). A comparative study of extraction of pectin from wet and dried peels using water based and microwave methods. *International Journal of Scientific and Engineering Research*.

[12] Yao W., Qiu H. M., Cheong K. L., Zhong S. (2022). Advances in anti-cancer effects and underlying mechanisms of marine algae polysaccharides. *International Journal of Biological*.

[13] Kamm B., Kamm M. (2007). Biorefineries–multi product processes. *White Biotechnology*.

[14] Aoac (1990). *Association of official analytical chemists*.

[15] Onyeike E. N., Oguike J. U. (2003). Influence of heat processing methods on the nutrient composition and lipid characterization of groundnut (Arachis hypogaea) seed pastes. *Biokemistri*.

[16] Fosu M. A., Ofori-Kwakye K., Kuntworbe N., Bonsu M. A. (2016). Investigation of blends of cashew and xanthan gums as a potential carrier for colonic delivery of ibuprofen. *International Journal of Pharm Tech Research*.

[17] Bonsu M. A., Ofori-Kwakye K., Kipo S. L., Boakye-Gyasi M. E., Fosu M. A. (2016). Development of oral dissolvable films of diclofenac sodium for osteoarthritis using Albizia and Khaya Gums as hydrophilic film formers. *Journal of drug delivery*.

[18] Pappas C. S., Malovikova A., Hromadkova Z., Tarantilis P. A., Ebringerova A., Polissiou M. G. (2004). Determination of the degree of esterification of pectinates with decyl and benzyl ester groups by diffuse reflectance infrared Fourier transform spectroscopy (DRIFTS) and curve-fitting deconvolution method. *Carbohydrate Polymers*.

[19] Güzel M., Akpınar Ö. (2019). Valorisation of fruit by-products: production characterization of pectins from fruit peels. *Food and Bioproducts Processing*.

[20] Evans W. C. (2009). *Trease and evans’ Pharmacognosy E-Book*.

[21] Monograph, Carbamazepine Extended-Release Tablets (2008). United States Pharmacopoeia and National Formulary.

[22] Adi-Dako O., Ofori-Kwakye K., Manso S. F., Boakye-Gyasi M. E., Sasu C., Pobee M. (2016). Physicochemical and antimicrobial properties of cocoa pod husk pectin intended as a versatile pharmaceutical excipient and nutraceutical. *Journal of Pharmaceutics*.

[23] Oppong E. E., Osei-Asare C. H., Klu M. W. (2016). Evaluation of the suspending properties of shea tree gum. *International Journal of Pharmacy and Pharmaceutical Sciences*.

[24] Nep E. I., Conway B. R. (2011). Evaluation of grewia polysaccharide gum as a suspending agent. *International Journal of Pharmacy and Pharmaceutical Sciences*.

[25] Mahmud H. S., Oyi A. R., Allagh T. S., Gwarzo M. S. (2010). Evaluation of the suspending property of Khaya snegalensis gum in co-trimoxazole suspensions. *Research Journal of Applied Sciences, Engineering and Technology*.

[26] Campbell M., Lee K. Y., Choo W. S. (2015). Extraction of pectin from watermelon rind. *Journal of Pharmacognosy & Natural Products*.

[27] Joel J. M., Barminas J. T., Riki E. Y., Yelwa J. M., Edeh F. (2018). Extraction and characterization of hydrocolloid pectin from goron tula (Azanza garckeana) fruit. *World Scientific News*.

[28] Pérez J., Gómez K., Vega L. (2022). Optimization and Preliminary Physicochemical Characterization of Pectin Extraction from Watermelon Rind (Citrullus lanatus) with Citric Acid. *International journal of food science*.

[29] Begum R., Aziz M. G., Uddin M. B., Yusof Y. A. (2014). Characterization of jackfruit (Artocarpus heterophyllus) waste pectin as influenced by various extraction conditions. *Agriculture and Agricultural Science Procedia*.

[30] Ooi D. J., Iqbal S., Ismail M. (2012). Proximate composition, nutritional attributes and mineral composition of Peperomia pellucida L.(Ketumpangan air) grown in Malaysia. *Molecules*.

[31] Hlaing T., Myint H. Z., Ngwe D. H. (2019). Studies on physicochemical properties and elemental analysis of citron and pomelo fruits peels pectins. *International Journal of Sciences: Basic and Applied Research*.

[32] Adjei F. K., Osei Y. A., Kuntworbe N., Ofori-Kwakye K. (2017). Evaluation of the disintegrant properties of native starches of five new cassava varieties in paracetamol tablet formulations. *Journal of Pharmaceutics*.

[33] Nandiyanto A. B. D., Oktiani R., Ragadhita R. (2019). How to read and interpret FTIR spectroscope of organic material. *Indonesian Journal of Science and Technology*.

[34] Muthukumaran C., Banupriya L., Harinee S. (2017). Pectin from muskmelon (Cucumis melo var. reticulatus) peels: extraction optimization and physicochemical properties. *3 Biotech*.

[35] Copikova J., Cerna M., Novotna M., Kaasova J. I. T. K. A., Synytsya A. (2013). Application of FT-IR spectroscopy in detection of food hydrocolloids confectionery jellies and in food supplements. *Czech Journal of Food Sciences*.

[36] The Lubrizol Corporation (2020). Paracetamol Suspension.

[37] Vázquez-Blanco S., González-Freire L., Dávila-Pousa M. C., Crespo-Diz C. (2018). pH determination as a quality standard for the elaboration of oral liquid compounding formula. *Farmacia Hospitalaria*.

[38] Nutan M. T., Reddy I. K. (2010). General principles of suspensions. *Pharmaceutical Suspensions*.

[39] Allen L., Ansel H. C. (2013). *Ansel’s Pharmaceutical Dosage Forms and Drug Delivery Systems*.

[40] Larsson M., Hill A., Duffy J. (2012). Suspension stability; why particle size, zeta potential and rheology are important. *Annual transactions of the Nordic rheology society*.

[41] Owusu F. W., Asare C. O., Enstie P. (2021). Formulation and In Vitro Evaluation of Oral Capsules and Suspension from the Ethanolic Extract of Cola nitida Seeds for the Treatment of Diarrhea. *BioMed Research international*.

[42] Bamigbola E. A., Olorode O. A., Uzim D. A. (2017). Evaluation of the suspending properties of Cola acuminata gum on calamine suspension. *Journal of Phytomedicine and Therapeutics*.

[43] Woldu G., Baymot B., Tesfay D., Demoz G. T. (2021). Evaluation of Aloe elegans Mucilage as a Suspending Agent in Paracetamol Suspension. *BioMed Research international*.

